# Deciphering the effect of phytosterols on Alzheimer’s disease and Parkinson’s disease: the mediating role of lipid profiles

**DOI:** 10.1186/s13195-024-01424-9

**Published:** 2024-03-09

**Authors:** Xingzhi Guo, Jing Yu, Rui Wang, Ning Peng, Rui Li

**Affiliations:** 1 Department of Geriatric Neurology, Shaanxi Provincial People’s Hospital, No. 256, Youyi West Road, Xi’an, Shaanxi 710068 China; 2 College of Life Sciences, Northwest University, Xi’an, Shaanxi 710069 China; 3 Shaanxi Provincial Key Laboratory of Infection and Immune Diseases, Shaanxi Provincial People’s Hospital, Xi’an, Shaanxi 710068 China; 4 Xi’an Key Laboratory of Stem Cell and Regenerative Medicine, Institute of Medical Research, Northwestern Polytechnical University, Xi’an, Shaanxi 710072 China

**Keywords:** Alzheimer's disease, Phytosterols, Lipid, Cholesterol, Mendelian randomization

## Abstract

**Background:**

Studies have suggested that blood circulating phytosterols, plant-derived sterols analogous to cholesterol, were associated with blood lipid levels and the risk of Alzheimer’s disease (AD) and Parkinson’s disease (PD). This Mendelian randomization (MR) study is performed to determine the causal effect of circulating phytosterols on AD and PD and evaluate the mediation effect of blood lipids.

**Methods:**

Leveraging genome-wide association studies summary-level data for phytosterols, blood lipids, AD, and PD, univariable and multivariable MR (MVMR) analyses were conducted. Four types of phytosterols (brassicasterol, campesterol, sitosterol, and stigmasterol), three blood lipids parameters (high-density lipoprotein cholesterol [HDL-C], non-HDL-C, and triglyceride), two datasets for AD and PD were used. Inverse-variance weighted method was applied as the primary analysis, and false discovery rate method was used for adjustment of multiple comparisons.

**Results:**

Using the largest AD dataset, genetically proxied higher levels of stigmasterol (OR = 0.593, 95%CI = 0.431–0.817, *P* = 0.004) and sitosterol (OR = 0.864, 95%CI = 0.791–0.943, *P* = 0.004) significantly correlated with a lower risk of AD. No significant associations were observed between all four types of phytosterols levels and PD. MVMR estimates showed that the above causal associations were missing after integrating the blood lipids as exposures. Sensitivity analyses confirmed the robustness of these associations, with no evidence of pleiotropy and heterogeneity.

**Conclusion:**

The study supports a potential beneficial role of blood stigmasterol and sitosterol in reducing the risk of AD, but not PD, which is dependent on modulating blood lipids. These insights highlight circulating stigmasterol and sitosterol as possible biomarkers and therapeutic targets for AD.

**Supplementary Information:**

The online version contains supplementary material available at 10.1186/s13195-024-01424-9.

## Introduction

Alzheimer’s disease (AD) and Parkinson’s disease (PD), the two most common neurodegenerative diseases, place a substantial burden to global public health systems [1–3]. Both AD and PD are characterized by the accumulation of misfolding peptides/proteins and progressive neuronal impairment in the brain, including beta-amyloid and p-tau for AD and alpha-synuclein for PD respectively [4]. Despite extensive efforts, the mechanisms driving the development of AD and PD remain not fully understood, and there is currently lack of effective treatment options to delay the progression of the disease [5]. Cholesterol is indispensable for maintaining neuronal membrane integrity, which is involved in synaptic plasticity and neuronal polarity [6, 7]. A large body of research indicates that dysregulation of cholesterol metabolism has been implicated in the pathogenesis of AD and PD [8, 9], which might serve as a potential therapeutic target for both conditions.

Phytosterols, plant-derived sterols, have been recognized for their role in regulating cholesterol homeostasis, immune response, and brain health [10, 11]. Due to similar structure to cholesterol, it is suggested that phytosterols might compete with dietary and biliary cholesterol absorption in the intestine to reduce blood cholesterol levels [12]. Additionally, previous studies showed that phytosterols also exert anti-inflammatory and antioxidant function [13], which were involved in the pathological changes in AD and PD. Increasing studies have suggested that circulating levels of phytosterols were associated the risk of AD and PD [14, 15]. However, since observational studies are often confounded by factors such as diet, lifestyle, lipids levels and medication use, the associations between phytosterols and AD and PD risk remain inconsistent [13, 16].

Mendelian randomization (MR) offers a method to minimize confounding inherent to observational studies by using genetic variants as instrumental variables (IVs) to infer causal relationships [17, 18]. We employ both univariable and multivariable MR approaches to investigate whether circulating phytosterols are causally linked to the risk of AD and PD, and to what extent this relationship is mediated by blood lipids profile.

## Materials and methods

### Study design and data

**Fig. 1:**
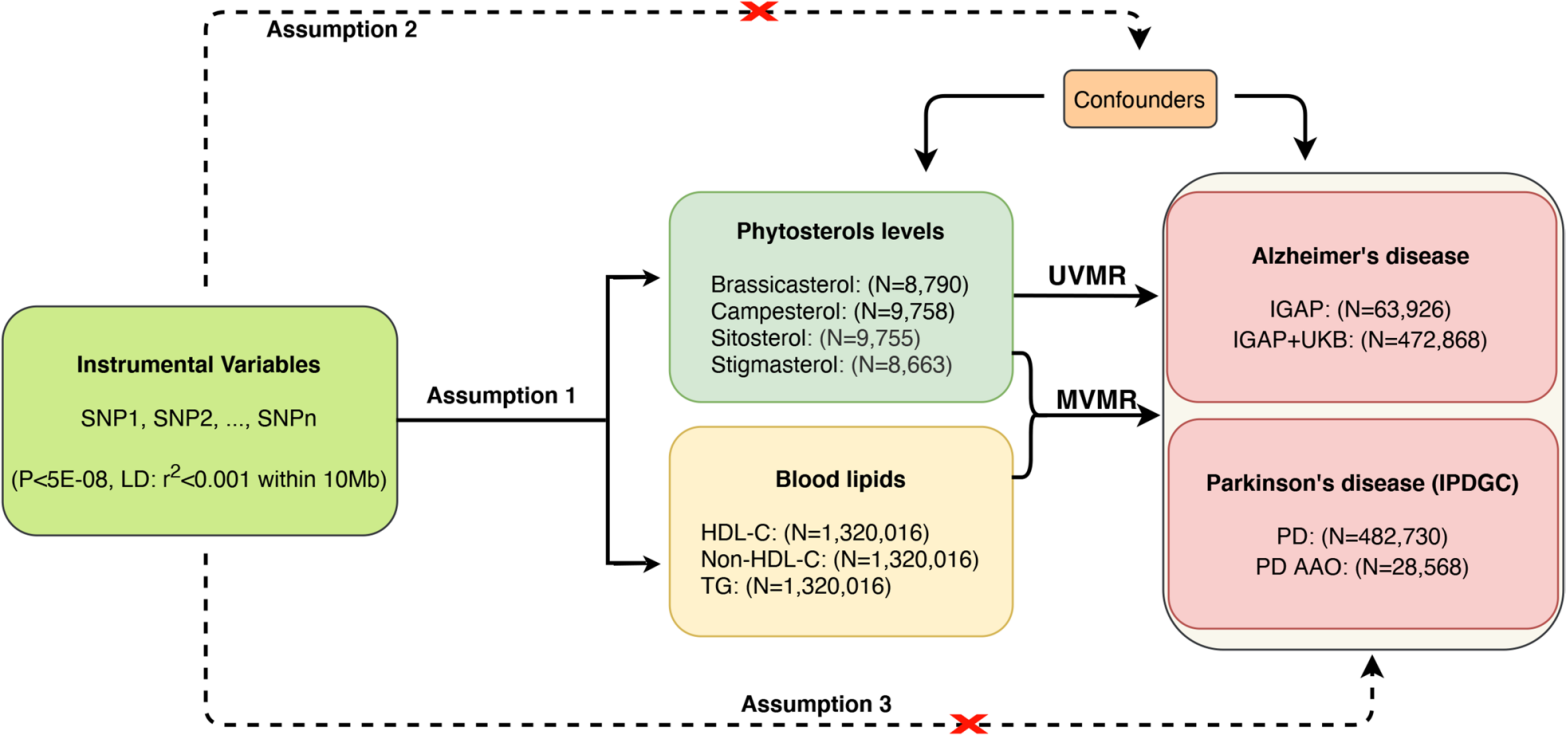
Flowchart for this study. The red cross sign means genetic variables not associated with confounding factors and outcomes. Assumption 1 indicates that SNPs are strongly associated with circulating phytosterols levels; Assumption 2 indicates that SNPs are not associated with confounding factors; Assumption 3 indicates that SNPs affect the risk of outcomes via circulating phytosterols levels directly. AD, Alzheimer’s disease; PD, Parkinson’s disease; PD AAO, Parkinson’s disease age at onset; SNP, single nucleotide polymorphism; LD, linkage disequilibrium; IPDGC, International Parkinson Disease Genomics Consortium; TG, triglycerides; HDL-C, high-density lipoprotein cholesterol; UVMR, univariable Mendelian randomization; MVMR, multivariable Mendelian randomization; UKB, UK Biobank; IGAP, International Genomics of Alzheimer’s Project; N, number

Univariable MR (UVMR) design was used to explore the causal impact of circulating phytosterols on the risk of AD and PD, while multivariable MR (MVMR) was applied to evaluate the mediating effect of blood lipids in the relationship between phytosterols and AD and PD (Fig. 1). This study was conducted utilizing publicly available genome-wide association studies (GWAS) summary-level data on phytosterols, blood lipids, AD, and PD of European decent (Table 1).

**Table 1:** GWAS summary-level data used in this study

Study	Author/Consortium	Year	Samples (N)	Population	PMID
**Phytosterols levels**					
Brassicasterol	Scholz et al.	2022	8,790	European	35,013,273
Campesterol	Scholz et al.	2022	9,758	European	35,013,273
Sitosterol	Scholz et al.	2022	9,755	European	35,013,273
Stigmasterol	Scholz et al.	2022	8,663	European	35,013,273
**Neurodegenerative diseases**					
Alzheimer’s disease (IGAP)	Kunkle et al.	2019	63,926	European	30,820,047
Alzheimer’s disease (IGAP + UKB)	Schwartzentruber et al.	2021	472,868	European	33,589,840
Parkinson’s disease (IPDGC)	Nalls et al.	2019	482,730	European	31,701,892
Parkinson’s disease age at oneset (IPDGC)	Blauwendraat et al.	2019	28,568	European	30,957,308
**Blood Lipids (GLGC)**^**a**^					
HDL cholesterol	Graham et al.	2023	1,320,016	European	37,237,109
Non-HDL cholesterol	Graham et al.	2023	1,320,016	European	37,237,109
Triglyceride	Graham et al.	2023	1,320,016	European	37,237,109

*IGAP *International Genomics of Alzheimer’s Project, *UKB *UK Biobank, *IPDGC *International Parkinson Disease Genomics Consortium, *GLGC *Global Lipids Genetics Consortium, *N *Number

^a^Summary-level data without UK Biobank participants

For phytosterols, four subtypes of phytosterols, including total brassicasterol (*N* = 8,790), campesterol (*N* = 9,758), sitosterol (*N* = 9,755), and stigmasterol (*N* = 8,663), were used in this study [19]. The serum concentrations of the above phytosterols were measured using the liquid chromatography tandem mass spectrometry [19]. For AD, one dataset was from the International Genomics of Alzheimer’s Project (IGAP) Consortium with 63,926 participants available [20], and another dataset were obtained from combined summary-level data on both IGAP and UK BioBank (UKB, *N* = 472,868) [21]. For PD, one dataset for PD (*N* = 482,730) [22] and another for PD age at onset (PD AAO, *N* = 28,568) [23] were from the International Parkinson Disease Genomics Consortium (IPDGC). For blood lipids as potential mediators, summary-level data on triglyceride (TG), high-density lipoprotein cholesterol (HDL-C), and non-HDL cholesterol (non-HDL-C, *N* = up to 1,320,016) were obtained from the latest large-scale GWAS meta-analysis by the Global Lipids Genetics Consortium (GLGC) [24]. The lipid levels were measured more than 8 h after fasting and were corrected for age, sex, and medication use. To avoid the impact from sample overlapping, only summary statistics without UKB samples were used for blood lipids [24]. The detailed information for each included study were well described in original studies (Table 1). Given that this study is based on anonymized summary-level data publicly available, no separated ethical approval and individual consent were required. The MR analysis was conducted adheres to the STROBE-MR guideline for MR study [25].

### Selection of instrumental variables

Genetic variants, namely single nucleotide polymorphisms in GWAS summary-level data, identified as IVs for circulating levels of each phytosterol should meet three principal assumptions [18]. First, IVs should pass the genome-wide significance threshold (*P* < 5E-08) and had no linkage disequilibrium (LD: *r*^2^ < 0.001 within 10 M bases). Second, IVs should not be associated with confounders related to both phytosterols levels and AD or PD. Third, IVs should affect the outcome indirectly through the exposure. In addition, the F-statistic value was calculated for each IV to avoid potential bias introduced by weaken IVs with F-statistic less than 10. Finally, a proxy SNP with *r*^2^ > 0.8 (LD) was used as a replacement for those IVs missing in corresponding outcome. A detailed list of IVs for each phytosterol was presented in Additional file 1: Table S1.

### Statistical analysis

The primary MR analysis was conducted using the inverse-variance weighted (IVW) method, which are confirmed in sensitivity analysis using MR-Egger, weighted median, weighted mode, simple median, and maximum likelihood approaches. To ensure the validity of MR estimates, the MR-Egger intercept test was used to detect directional pleiotropy [26] and the MR pleiotropy residual sum and outlier (MR-PRESSO) test was applied to identify and correct for outliers that could bias the results [27]. To validate the robustness of our MR estimates, the heterogeneity was assessed using the Cochran’s Q statistic test. The MVMR analysis was performed to evaluate the mediating effect of blood lipids in the relationship between phytosterols and AD and PD by treating both phytosterols and blood lipids as exposures simultaneously. All analyses were performed using the TwoSampleMR (0.5.6) package in R statistical software [28]. *P*-values were adjusted for multiple testing using the false discovery rate (FDR) method for each outcome and an adjusted P-value less than 0.05 was considered significant.

## Results

In summary, there were four, three, six, and four valid IVs for total brassicasterol, campesterol, sitosterol, and stigmasterol respectively. The F-statistics value for all IVs were above the threshold of 10, indicating a low risk of weak instrument bias.

### UVMR analysis

**Fig. 2:**
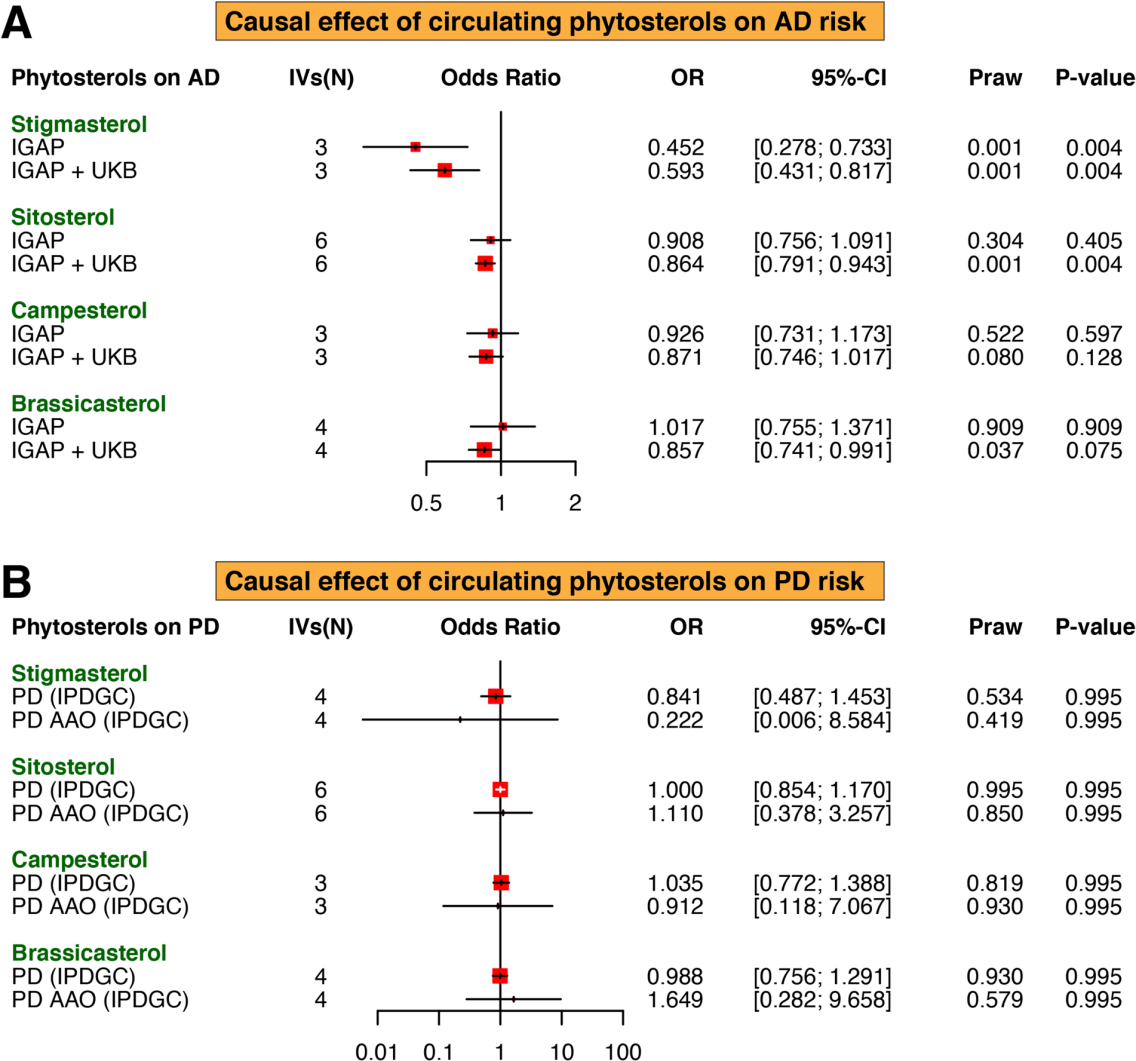
Effects of circulating phytosterols levels on the risk of Alzheimer’s disease and Parkinson’s disease. Using two different summary-level data on AD, genetically predicted circulating levels of stigmasterol and sitosterol were associated with a decreased risk of AD (**A**). There was no effect of phytosterols levels on the risk of PD and age at onset of PD (**B**). Alzheimer’s disease; PD, Parkinson’s disease; PD OAA, PD age at onset; UKB, UK Biobank; SNP, single nucleotide polymorphism; OR, odds ratio; IGAP, International Genomics of Alzheimer’s Project; IVs, instrumental variables; N, number

**Fig. 3:**
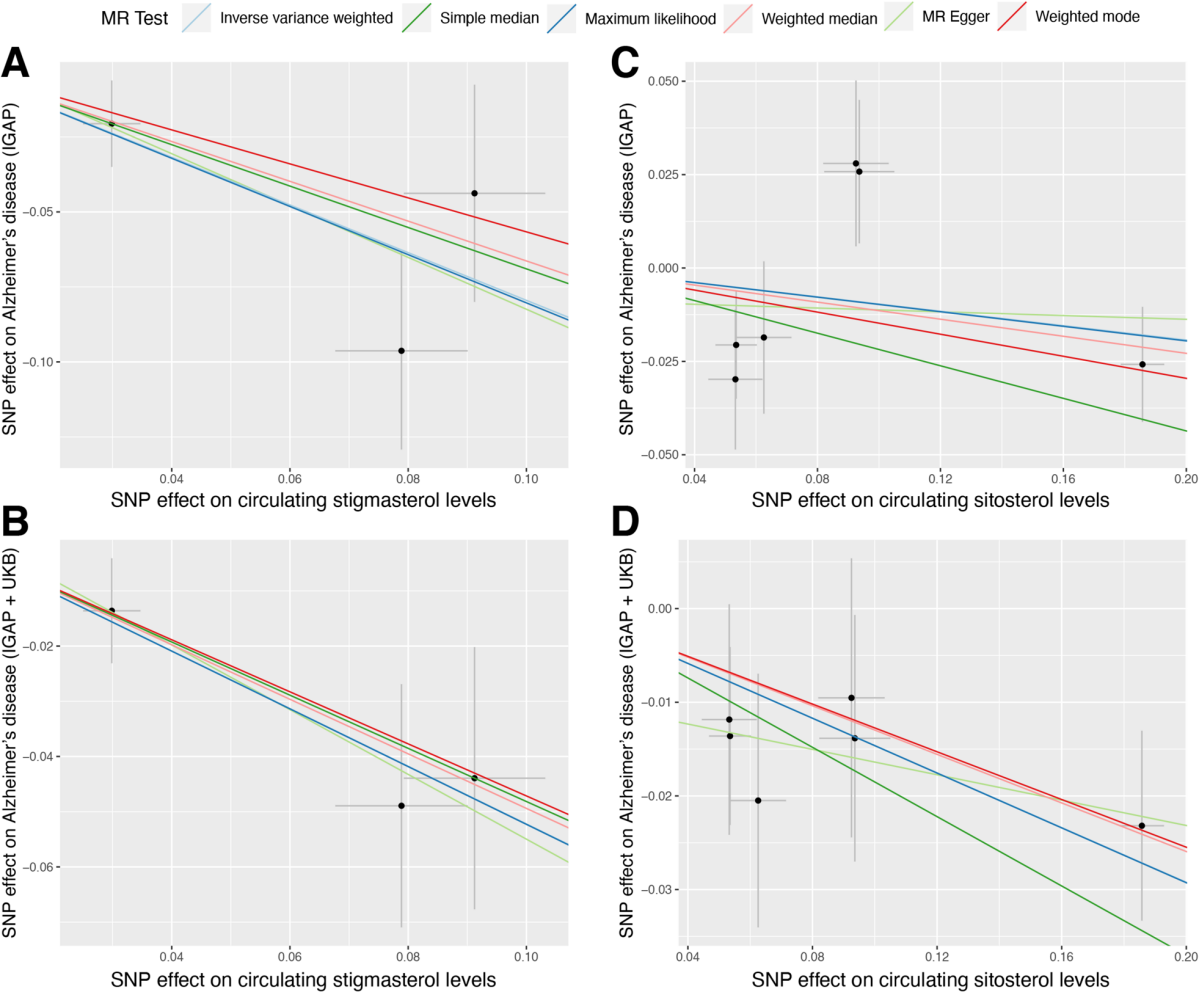
Scatter plots for the causal effect of circulating stigmasterol and sitosterol levels and Alzheimer’s disease. **A** and **B** showed the SNPs’ effect on circulating stigmasterol levels and AD using summary-level data from IGAP and IGAP + UKB, respectively. **C** and **D** showed the SNPs’ effect on circulating sitosterol levels and AD using summary-level data from IGAP and IGAP + UKB, respectively. The slope indicated the causal estimates for each method. AD, Alzheimer’s disease; MR, Mendelian randomization; SNP, single nucleotide polymorphism; UKB, UK Biobank; IGAP, International Genomics of Alzheimer’s Project

Using the IVW approach, genetically determined higher circulating stigmasterol levels were significantly associated with a reduced risk of AD (IGAP: odds ratio [OR] = 0.452, 95%CI = 0.278–0.733, *P* = 0.004; IGAP + UKB: OR = 0.593, 95%CI = 0.431–0.817, *P* = 0.004). Additionally, a significant inverse relationship was found between sitosterol levels and AD using the IGAP + UKB dataset (OR = 0.864, 95%CI = 0.791–0.943, *P* = 0.004), but not replicated in the IGAP dataset. However, there were no significant causal effects of circulating levels of campesterol and brassicasterol on the risk of AD (Fig. 2A). Moreover, no significant relationships were observed between all four phytosterols subtypes and the risk of PD or PD age one set (Fig. 2B). Estimates from other MR approaches showed similar trend to the primary analysis (Additional file 1: Table S2), as displayed in the scatter plots (Fig. 3). This finding suggests a potential protective effect of phytosterols against AD. The MR-Egger regression did not indicate the presence of directional pleiotropy (Table 2). No obvious outlier(s) was found in the MR-PRESSO test (Table 2) and the leave-one-out plots showed good stability of the estimates (Additional file 1: Figure S1).

**Table 2:** Heterogeneity and pleiotropy test between blood phytosterols levels, Alzheimer's disease, and Parkinson's disease

Exposure	Outcome	IVs (N)	Cochran Q test		MR-Egger Intercept (P)	MR-PRESSO RSSobs (P)^a^
			MR-Egger (P)	IVW (P)		
**Stigmasterol**	AD (IGAP)	3	1.697 (0.193)	1.720 (0.423)	0.0040 (0.925)	-
	AD (IGAP + UKB)	3	0.145 (0.703)	0.192 (0.909)	0.0037 (0.865)	-
	PD (IPDGC)	4	0.654 (0.721)	0.661 (0.882)	0.0027 (0.944)	1.259 (0.873)
	PD AAO (IPDGC)	4	1.839 (0.399)	1.846 (0.605)	-0.0188 (0.940)	3.157 (0.645)
**Sitosterol**	AD (IGAP)	6	9.235 (0.055)	9.568 (0.088)	-0.0087 (0.723)	13.369 (0.239)
	AD (IGAP + UKB)	6	0.472 (0.976)	1.406 (0.924)	-0.0096 (0.388)	2.843 (0.888)
	PD (IPDGC)	6	3.346 (0.502)	3.432 (0.634)	0.0054 (0.783)	3.763 (0.778)
	PD AAO (IPDGC)	6	2.015 (0.733)	2.029 (0.845)	-0.0151 (0.909)	2.507 (0.897)
**Campesterol**	AD (IGAP)	3	0.007 (0.932)	0.393 (0.821)	-0.0122 (0.646)	-
	AD (IGAP + UKB)	3	0.110 (0.740)	0.506 (0.777)	-0.0082 (0.643)	-
	PD (IPDGC)	3	0.465 (0.495)	0.618 (0.734)	-0.0099 (0.763)	-
	PD AAO (IPDGC)	3	2.129 (0.144)	2.136 (0.344)	-0.0139 (0.964)	-
**Brassicasterol**	AD (IGAP)	4	4.612 (0.100)	5.412 (0.144)	0.0153 (0.615)	15.931 (0.302)
	AD (IGAP + UKB)	4	0.553 (0.758)	3.061 (0.382)	0.0179 (0.254)	9.708 (0.435)
	PD (IPDGC)	4	1.244 (0.537)	1.408 (0.704)	0.0089 (0.725)	1.711 (0.832)
	PD AAO (IPDGC)	4	0.856 (0.652)	1.837 (0.607)	-0.1438 (0.426)	2.417 (0.704)

*AD *Alzheimer’s disease, *PD* Parkinson’s disease, *IGAP* International Genomics of Alzheimer’s Project, *UKB* UK Biobank, *IPDGC* International Parkinson Disease Genomics Consortium, *AAO* Age at oneset, *IVs* Instrumental variables, *MR-PRESSO* Mendelian Randomization Pleiotropy RESidual Sum and Outlier, *P P*-value, *N* Number

^a^Not available for those exposure with less than 4 valid instrumental variables

### MVMR analysis

**Fig. 4:**
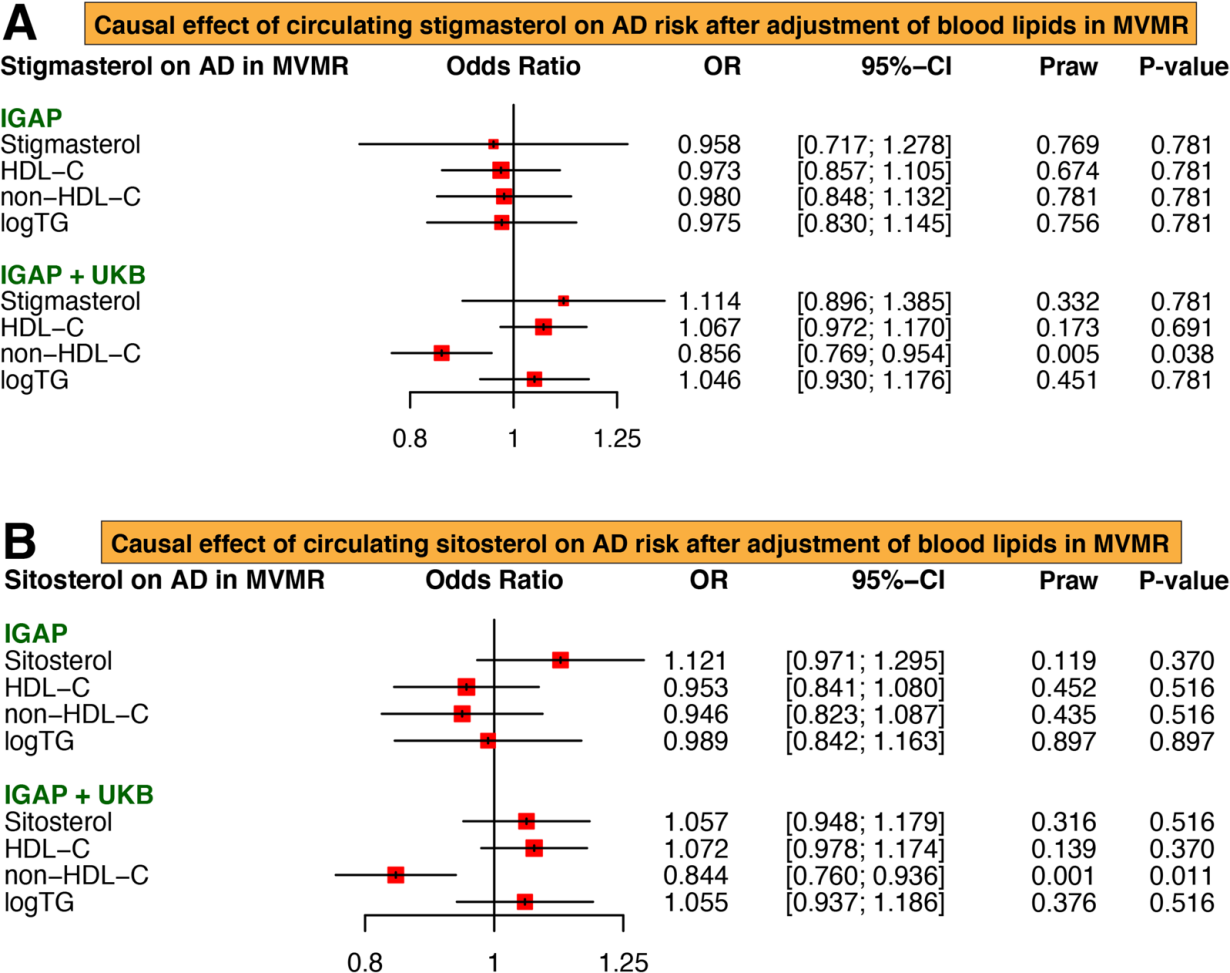
Effects of circulating stigmasterol and sitosterol levels on the risk of Alzheimer’s disease in multivariable Mendelian randomization. The causal effect of circulating stigmasterol (**A**) and sitosterol (**B**) on AD disappeared after adjustment of blood lipids in MVMR analysis. AD, Alzheimer’s disease; HDL-C, high-density lipoprotein cholesterol; TG, triglycerides; OR, odds ratio; MVMR, multivariable Mendelian randomization; UKB, UK Biobank; IGAP, International Genomics of Alzheimer’s Project

Since UVMR showed that circulating levels of stigmasterol and sitosterol were only significantly associated with AD, but not PD, we performed a MVMR analysis to evaluate the potential role of blood lipids in the relationship between these two phytosterols and AD. By using either stigmasterol or sitosterol and blood lipids as exposures, the estimates showed that the impact of stigmasterol and sitosterol on AD was loss (*P* > 0.05) (Fig. 4). Among the HDL-C, non-HDL-C, and triglyceride, only non-HDL-C showed a significantly inverse association with AD using the IGAP + UKB dataset (Stigmasterol: OR = 0.856, 95%CI = 0.769–0.954, *P* = 0.038; Sitosterol: OR = 0.844, 95%CI = 0.760–0.936, *P* = 0.011). These data suggested that the protective effect of stigmasterol and sitosterol on AD may be potentially mediated by their impact on blood lipid profiles, especially on non-HDL-C levels.

## Discussion

The present study provides evidence supporting an inverse relationship between circulating levels of stigmasterol and sitosterol and the risk of AD, but not PD. In addition, MVMR analysis reveals that blood lipids, especially for non-HDL-C, may serve as a potential mediator in the relationship between phytosterols and AD risk. Our findings underscore the importance of considering the phytosterols (stigmasterol and sitosterol) in AD risk assessment and intervention strategies.

There are four common subtypes of phytosterols used in this MR study, but the results showed that only stigmasterol and sitosterol levels were significantly associated with a decreased risk of AD. It is worth noting that the OR estimates for campesterol and brassicasterol on AD was also smaller than one, despite lacking statistical significance. These results suggested that the protective effect of phytosterols on AD might be a class effect rather than an action limited to individual compounds. However, as both campesterol and brassicasterol are derived from sitosterol after removing the 24th carbon atom [29], it cannot be ruled out that the slight differences in their chemical structures might lead to different functions in AD. Thus, further studies remain deserved to assess whether there is specificity of the protective effect across different phytosterols subtypes.

Numerous studies have suggested a relationship between lipids metabolism disorder and the risk of neurodegenerative diseases [30–32], but the role of phytosterols in AD and PD was far from conclusive [13, 16, 33]. As phytosterols could reduce intestinal cholesterol absorption and blood cholesterol levels, it generally believed that phytosterols may exert a protective effect against AD and PD [11, 34]. Additionally, previous animal studies have demonstrated that phytosterols exerted a neuroprotective role via inhibiting inflammatory response and oxidative stress, which were tightly implicated in the pathogenesis of both AD and PD [13]. Our MR study revealed that blood phytosterols levels were only significantly associated with a decreased risk of AD, but not PD. The potential reason contributing to the inconsistent impact of phytosterols on AD and PD may stem from differing sensitivities to blood LDL-C status [35]. For example, it is well established that apolipoprotein E4 (APOE4), a crucial lipoprotein crucial for lipid homeostasis, plays a more significant role in AD compared to PD [36–38], suggesting that AD may be particularly vulnerable to disturbances in lipid metabolism.

Our MVMR analysis showed that the protective effect of phytosterols on AD were dependent on blood lipids. A recent cohort study with more than 86,000 older adults showed that individuals with higher triglyceride levels had a reduced risk of cognitive decline and dementia compared to those with lower triglyceride levels [39]. In addition, another study on more than 80 triglyceride species indicated that serum polyunsaturated fatty acid-containing triglycerides were decreased in patients with mild cognitive impairment or AD, and were significantly linked to hippocampal volume and entorhinal cortical thickness [40]. These data suggested that triglyceride might mediate the effect of phytosterols on AD, but no significant relationship between TG levels and AD was observed in our MVMR analysis. Moreover, previous studies have showed that higher levels of non–HDL-C were related with a reduced risk of AD [41]. Our MVMR results support the concept that non-HDL-C levels was causally associated with a decreased risk of AD, even after adjustment of HDL-C and TG. However, additional studies showed that individuals aged 60–79 years with low and high non-HDL-C levels (120 mg/dl < or > 210 mg/dl) had a higher risk of AD than those with intermediate levels (160 mg/dl) [42], suggesting a nonlinear relationship (U shape) of non-HDL-C levels on AD [43]. Further research is needed to elucidate the underlying biological mechanisms by which non-HDL mediates the protective effect of phytosterols on AD.

### Strength and limitation

Our study used two different AD GWAS datasets to assess the relationship between phytosterols and AD and obtained consistent results. In addition, the MR estimates had the same trend across different MR methods in sensitivity analyses, suggesting good stability. Moreover, no significant pleiotropy and outlier(s) was identified by MR-Egger intercept and MR-PRESSO test respectively, supporting the validity of the causal inference. However, there are several limitations to be addressed. First, less than 10 IVs were available for each phytosterols subtypes, which might weaken the estimates between phytosterols levels and AD or PD. Second, although several approaches were used to validate the MR assumptions, we cannot fully exclude all possible violations of these assumptions, such as undetected pleiotropy. Third, since the present MR study was based on participants of European ancestry, whether this association exists in other population still need to be further elucidated. Fourth, due to only summary-level data available, we could not rule out whether there was a non-linear association between phytosterols levels and AD or PD. Fifth, the inverse relationship between sitosterol levels and AD was only observed in the IGAP + UKB dataset, but not in the IGAP dataset, which might be due to the smaller sample size in IGAP (*N* = 63,926) compared to IGAP + UKB (*N* = 472,868). Further studies with a larger sample size are warranted to confirm these findings.

## Conclusion

The present MR study reveals that phytosterols play a protective role against AD, but not PD. In addition, MVMR analysis indicates that the beneficial role of phytosterols on AD is mainly mediated by blood lipids, especially non-HDL-C, suggesting that dietary interventions to increase phytosterol intake could be potential strategies for AD prevention. Future studies like randomized controlled trials are warrant to confirm the causality and effectiveness of phytosterol-rich diets or supplements in reducing AD risk and translating these findings into practical dietary guidelines.

### Supplementary Information

**Supplementary Material 1.**

## Data Availability

No datasets were generated or analysed during the current study.

## Abbreviations

AD: Alzheimer’s disease
APOE4: Apolipoprotein E4
CI: Confidence interval
FDR: False discovery rate
GLGC: Global Lipids Genetics Consortium
GWAS: Genome-wide association studies
HDL-C: High-density lipoprotein cholesterol
IGAP: International Genomics of Alzheimer’s Project
IPDGC: International Parkinson Disease Genomics Consortium
IVs: Instrumental variables
IVW: Inverse-variance weighted
LD: Linkage disequilibrium
MR: Mendelian randomization
MR-PRESSO: MR-Pleiotropy RESidual Sum and Outlier
MVMR: Multivariable Mendelian randomization
OR: Odds ratio
PD: Parkinson’s disease
SNPs: Single nucleotide polymorphisms
TG: Triglyceride
UKB: UK BioBank
UVMR: Univariable Mendelian randomization
