# Supplementary material for: Deciphering the effect of phytosterols on Alzheimer’s disease and Parkinson’s disease: the mediating role of lipid profiles
Source: Alzheimers Res Ther. 2024 Mar 9;16:53. doi: 10.1186/s13195-024-01424-9 (PMC10924343; doi:10.1186/s13195-024-01424-9)
Supplement: Supplementary file 1 — Supplementary Material 1. [file 13195_2024_1424_MOESM1_ESM.docx]

**Deciphering the Effect of Phytosterols on Alzheimer's Disease and Parkinson’s disease: the Mediating Role of Lipid Profiles**

*There were 2 supplementary Tables and 1 supplementary Figure in this study.*

Supplementary Table S1. Independent instrumental variables valid for each exposure used in this study.

| **Phytosterols** | **SNP** | **EA** | **OA** | **EAF** | **BETA** | **SE** | **P-value** | **R^2^** | **F-statistic** |
| --- | --- | --- | --- | --- | --- | --- | --- | --- | --- |
| Stigmasterol | rs138958276 | A | G | 0.0439 | -0.0912 | 0.0120 | 2.38E-14 | 0.0067 | 58.0929 |
| Stigmasterol | rs2286779 | C | G | 0.5288 | 0.0299 | 0.0048 | 7.09E-10 | 0.0044 | 37.9694 |
| Stigmasterol | rs72798830 | T | C | 0.0462 | 0.0789 | 0.0112 | 1.74E-12 | 0.0057 | 49.6274 |
| Stigmasterol | rs7412 | T | C | 0.0867 | -0.0578 | 0.0093 | 5.73E-10 | 0.0044 | 38.4117 |
| Sitosterol | rs10846744 | C | G | 0.1705 | 0.0626 | 0.0090 | 2.91E-12 | 0.0050 | 48.6167 |
| Sitosterol | rs17725246 | C | T | 0.2032 | 0.0533 | 0.0088 | 1.46E-09 | 0.0037 | 36.5298 |
| Sitosterol | rs185263492 | G | T | 0.1549 | 0.0936 | 0.0114 | 2.46E-16 | 0.0097 | 67.2660 |
| Sitosterol | rs2286779 | C | G | 0.5338 | 0.0535 | 0.0067 | 1.92E-15 | 0.0064 | 63.1366 |
| Sitosterol | rs4299376 | T | G | 0.6823 | -0.1857 | 0.0071 | 1.48E-151 | 0.0659 | 687.7024 |
| Sitosterol | rs6709904 | G | A | 0.1090 | -0.0925 | 0.0107 | 4.00E-18 | 0.0077 | 75.3208 |
| Campesterol | rs217385 | T | G | 0.4312 | -0.0415 | 0.0053 | 6.34E-15 | 0.0062 | 60.7301 |
| Campesterol | rs2519093 | T | C | 0.2153 | 0.0446 | 0.0063 | 1.59E-12 | 0.0051 | 49.8825 |
| Campesterol | rs6544716 | G | T | 0.7308 | -0.1211 | 0.0058 | 6.73E-97 | 0.0427 | 435.6837 |
| Brassicasterol | rs11887534 | C | G | 0.0637 | -0.2059 | 0.0121 | 4.34E-65 | 0.0319 | 289.9723 |
| Brassicasterol | rs4953029 | A | C | 0.2309 | -0.0426 | 0.0071 | 1.99E-09 | 0.0041 | 35.9655 |
| Brassicasterol | rs6730449 | A | T | 0.2458 | 0.0467 | 0.0070 | 1.87E-11 | 0.0051 | 45.0040 |
| Brassicasterol | rs7590687 | C | T | 0.9237 | -0.1195 | 0.0120 | 2.59E-23 | 0.0111 | 98.7761 |

Note: EA, effect allele; OA, other allele; EAF, effect allele frequency; SE, standard error; SNP, number of single nucleotide polymorphism.

Supplementary Table S2. Estimates of circulating phytosterols levels on AD and PD using different statistical approaches.

| **Exposure** | **Outcome** | **Method** | **IVs(N)** | **Odds ratio (95%CI)** | **P-value** |
| --- | --- | --- | --- | --- | --- |
| Stigmasterol | AD (IGAP) | Inverse variance weighted | 3 | 0.452 (0.278 to 0.733) | 0.001 |
| Stigmasterol | AD (IGAP) | Maximum likelihood | 3 | 0.448 (0.271 to 0.741) | 0.002 |
| Stigmasterol | AD (IGAP) | MR Egger | 3 | 0.421 (0.114 to 1.565) | 0.420 |
| Stigmasterol | AD (IGAP) | Simple median | 3 | 0.502 (0.259 to 0.971) | 0.041 |
| Stigmasterol | AD (IGAP) | Weighted median | 3 | 0.515 (0.277 to 0.959) | 0.037 |
| Stigmasterol | AD (IGAP) | Weighted mode | 3 | 0.568 (0.264 to 1.222) | 0.285 |
| Stigmasterol | AD (IGAP + UKB) | Inverse variance weighted | 3 | 0.593 (0.431 to 0.817) | 0.001 |
| Stigmasterol | AD (IGAP + UKB) | Maximum likelihood | 3 | 0.593 (0.426 to 0.826) | 0.002 |
| Stigmasterol | AD (IGAP + UKB) | MR Egger | 3 | 0.556 (0.286 to 1.082) | 0.334 |
| Stigmasterol | AD (IGAP + UKB) | Simple median | 3 | 0.618 (0.412 to 0.927) | 0.020 |
| Stigmasterol | AD (IGAP + UKB) | Weighted median | 3 | 0.610 (0.413 to 0.901) | 0.013 |
| Stigmasterol | AD (IGAP + UKB) | Weighted mode | 3 | 0.624 (0.404 to 0.965) | 0.168 |
| Stigmasterol | PD (IPDGC) | Inverse variance weighted | 4 | 0.841 (0.487 to 1.453) | 0.534 |
| Stigmasterol | PD (IPDGC) | Maximum likelihood | 4 | 0.840 (0.485 to 1.455) | 0.534 |
| Stigmasterol | PD (IPDGC) | MR Egger | 4 | 0.802 (0.224 to 2.873) | 0.767 |
| Stigmasterol | PD (IPDGC) | MR_PRESSO Outlier-corrected | 4 | NA (NA to NA) | NA |
| Stigmasterol | PD (IPDGC) | MR_PRESSO Raw | 4 | 0.841 (0.651 to 1.087) | 0.277 |
| Stigmasterol | PD (IPDGC) | Simple median | 4 | 0.783 (0.421 to 1.458) | 0.440 |
| Stigmasterol | PD (IPDGC) | Weighted median | 4 | 0.782 (0.419 to 1.458) | 0.438 |
| Stigmasterol | PD (IPDGC) | Weighted mode | 4 | 0.753 (0.333 to 1.700) | 0.543 |
| Stigmasterol | PD AAO (IPDGC) | Inverse variance weighted | 4 | 0.222 (0.006 to 8.584) | 0.419 |
| Stigmasterol | PD AAO (IPDGC) | Maximum likelihood | 4 | 0.219 (0.006 to 8.645) | 0.418 |
| Stigmasterol | PD AAO (IPDGC) | MR Egger | 4 | 0.308 (0.000 to 1346.692) | 0.809 |
| Stigmasterol | PD AAO (IPDGC) | MR_PRESSO Outlier-corrected | 4 | NA (NA to NA) | NA |
| Stigmasterol | PD AAO (IPDGC) | MR_PRESSO Raw | 4 | 0.222 (0.013 to 3.905) | 0.379 |
| Stigmasterol | PD AAO (IPDGC) | Simple median | 4 | 0.408 (0.005 to 33.150) | 0.689 |
| Stigmasterol | PD AAO (IPDGC) | Weighted median | 4 | 0.390 (0.005 to 28.335) | 0.667 |
| Stigmasterol | PD AAO (IPDGC) | Weighted mode | 4 | 0.491 (0.001 to 165.710) | 0.826 |
| Sitosterol | AD (IGAP) | Inverse variance weighted | 6 | 0.908 (0.756 to 1.091) | 0.304 |
| Sitosterol | AD (IGAP) | Maximum likelihood | 6 | 0.907 (0.793 to 1.037) | 0.153 |
| Sitosterol | AD (IGAP) | MR Egger | 6 | 0.975 (0.641 to 1.483) | 0.912 |
| Sitosterol | AD (IGAP) | MR_PRESSO Outlier-corrected | 6 | NA (NA to NA) | NA |
| Sitosterol | AD (IGAP) | MR_PRESSO Raw | 6 | 0.908 (0.756 to 1.091) | 0.351 |
| Sitosterol | AD (IGAP) | Simple median | 6 | 0.804 (0.635 to 1.018) | 0.070 |
| Sitosterol | AD (IGAP) | Weighted median | 6 | 0.892 (0.765 to 1.041) | 0.146 |
| Sitosterol | AD (IGAP) | Weighted mode | 6 | 0.863 (0.727 to 1.024) | 0.152 |
| Sitosterol | AD (IGAP + UKB) | Inverse variance weighted | 6 | 0.864 (0.791 to 0.943) | 0.001 |
| Sitosterol | AD (IGAP + UKB) | Maximum likelihood | 6 | 0.864 (0.791 to 0.944) | 0.001 |
| Sitosterol | AD (IGAP + UKB) | MR Egger | 6 | 0.935 (0.779 to 1.121) | 0.506 |
| Sitosterol | AD (IGAP + UKB) | MR_PRESSO Outlier-corrected | 6 | NA (NA to NA) | NA |
| Sitosterol | AD (IGAP + UKB) | MR_PRESSO Raw | 6 | 0.864 (0.825 to 0.905) | 0.002 |
| Sitosterol | AD (IGAP + UKB) | Simple median | 6 | 0.831 (0.721 to 0.958) | 0.011 |
| Sitosterol | AD (IGAP + UKB) | Weighted median | 6 | 0.878 (0.794 to 0.971) | 0.011 |
| Sitosterol | AD (IGAP + UKB) | Weighted mode | 6 | 0.880 (0.792 to 0.978) | 0.064 |
| Sitosterol | PD (IPDGC) | Inverse variance weighted | 6 | 1.000 (0.854 to 1.170) | 0.995 |
| Sitosterol | PD (IPDGC) | Maximum likelihood | 6 | 1.000 (0.853 to 1.171) | 0.995 |
| Sitosterol | PD (IPDGC) | MR Egger | 6 | 0.957 (0.689 to 1.330) | 0.807 |
| Sitosterol | PD (IPDGC) | MR_PRESSO Outlier-corrected | 6 | NA (NA to NA) | NA |
| Sitosterol | PD (IPDGC) | MR_PRESSO Raw | 6 | 1.000 (0.877 to 1.139) | 0.994 |
| Sitosterol | PD (IPDGC) | Simple median | 6 | 0.951 (0.741 to 1.222) | 0.697 |
| Sitosterol | PD (IPDGC) | Weighted median | 6 | 0.985 (0.833 to 1.165) | 0.863 |
| Sitosterol | PD (IPDGC) | Weighted mode | 6 | 0.997 (0.826 to 1.205) | 0.980 |
| Sitosterol | PD AAO (IPDGC) | Inverse variance weighted | 6 | 1.110 (0.378 to 3.257) | 0.850 |
| Sitosterol | PD AAO (IPDGC) | Maximum likelihood | 6 | 1.110 (0.378 to 3.261) | 0.850 |
| Sitosterol | PD AAO (IPDGC) | MR Egger | 6 | 1.254 (0.133 to 11.799) | 0.853 |
| Sitosterol | PD AAO (IPDGC) | MR_PRESSO Outlier-corrected | 6 | NA (NA to NA) | NA |
| Sitosterol | PD AAO (IPDGC) | MR_PRESSO Raw | 6 | 1.110 (0.559 to 2.203) | 0.778 |
| Sitosterol | PD AAO (IPDGC) | Simple median | 6 | 0.913 (0.179 to 4.655) | 0.913 |
| Sitosterol | PD AAO (IPDGC) | Weighted median | 6 | 1.245 (0.388 to 3.994) | 0.713 |
| Sitosterol | PD AAO (IPDGC) | Weighted mode | 6 | 1.222 (0.346 to 4.317) | 0.768 |
| Campesterol | AD (IGAP) | Inverse variance weighted | 3 | 0.926 (0.731 to 1.173) | 0.522 |
| Campesterol | AD (IGAP) | Maximum likelihood | 3 | 0.926 (0.731 to 1.173) | 0.522 |
| Campesterol | AD (IGAP) | MR Egger | 3 | 1.064 (0.646 to 1.751) | 0.848 |
| Campesterol | AD (IGAP) | Simple median | 3 | 0.807 (0.534 to 1.220) | 0.309 |
| Campesterol | AD (IGAP) | Weighted median | 3 | 0.925 (0.726 to 1.178) | 0.527 |
| Campesterol | AD (IGAP) | Weighted mode | 3 | 0.962 (0.736 to 1.258) | 0.803 |
| Campesterol | AD (IGAP + UKB) | Inverse variance weighted | 3 | 0.871 (0.746 to 1.017) | 0.080 |
| Campesterol | AD (IGAP + UKB) | Maximum likelihood | 3 | 0.871 (0.745 to 1.017) | 0.081 |
| Campesterol | AD (IGAP + UKB) | MR Egger | 3 | 0.955 (0.689 to 1.324) | 0.828 |
| Campesterol | AD (IGAP + UKB) | Simple median | 3 | 0.854 (0.664 to 1.099) | 0.221 |
| Campesterol | AD (IGAP + UKB) | Weighted median | 3 | 0.883 (0.747 to 1.044) | 0.146 |
| Campesterol | AD (IGAP + UKB) | Weighted mode | 3 | 0.889 (0.749 to 1.056) | 0.313 |
| Campesterol | PD (IPDGC) | Inverse variance weighted | 3 | 1.035 (0.772 to 1.388) | 0.819 |
| Campesterol | PD (IPDGC) | Maximum likelihood | 3 | 1.035 (0.772 to 1.388) | 0.819 |
| Campesterol | PD (IPDGC) | MR Egger | 3 | 1.155 (0.618 to 2.160) | 0.730 |
| Campesterol | PD (IPDGC) | Simple median | 3 | 1.068 (0.671 to 1.702) | 0.781 |
| Campesterol | PD (IPDGC) | Weighted median | 3 | 1.063 (0.783 to 1.443) | 0.694 |
| Campesterol | PD (IPDGC) | Weighted mode | 3 | 1.073 (0.776 to 1.484) | 0.711 |
| Campesterol | PD AAO (IPDGC) | Inverse variance weighted | 3 | 0.912 (0.118 to 7.067) | 0.930 |
| Campesterol | PD AAO (IPDGC) | Maximum likelihood | 3 | 0.912 (0.125 to 6.637) | 0.928 |
| Campesterol | PD AAO (IPDGC) | MR Egger | 3 | 1.067 (0.002 to 493.069) | 0.987 |
| Campesterol | PD AAO (IPDGC) | Simple median | 3 | 0.997 (0.065 to 15.260) | 0.998 |
| Campesterol | PD AAO (IPDGC) | Weighted median | 3 | 1.001 (0.117 to 8.545) | 0.999 |
| Campesterol | PD AAO (IPDGC) | Weighted mode | 3 | 1.063 (0.112 to 10.078) | 0.962 |
| Brassicasterol | AD (IGAP) | Inverse variance weighted | 4 | 1.017 (0.755 to 1.371) | 0.909 |
| Brassicasterol | AD (IGAP) | Maximum likelihood | 4 | 1.018 (0.814 to 1.272) | 0.878 |
| Brassicasterol | AD (IGAP) | MR Egger | 4 | 0.889 (0.508 to 1.557) | 0.722 |
| Brassicasterol | AD (IGAP) | MR_PRESSO Outlier-corrected | 4 | NA (NA to NA) | NA |
| Brassicasterol | AD (IGAP) | MR_PRESSO Raw | 4 | 1.017 (0.755 to 1.371) | 0.916 |
| Brassicasterol | AD (IGAP) | Simple median | 4 | 1.123 (0.789 to 1.599) | 0.519 |
| Brassicasterol | AD (IGAP) | Weighted median | 4 | 0.865 (0.672 to 1.115) | 0.263 |
| Brassicasterol | AD (IGAP) | Weighted mode | 4 | 0.865 (0.646 to 1.157) | 0.400 |
| Brassicasterol | AD (IGAP + UKB) | Inverse variance weighted | 4 | 0.857 (0.741 to 0.991) | 0.037 |
| Brassicasterol | AD (IGAP + UKB) | Maximum likelihood | 4 | 0.856 (0.741 to 0.990) | 0.036 |
| Brassicasterol | AD (IGAP + UKB) | MR Egger | 4 | 0.734 (0.577 to 0.933) | 0.127 |
| Brassicasterol | AD (IGAP + UKB) | MR_PRESSO Outlier-corrected | 4 | NA (NA to NA) | NA |
| Brassicasterol | AD (IGAP + UKB) | MR_PRESSO Raw | 4 | 0.857 (0.741 to 0.991) | 0.129 |
| Brassicasterol | AD (IGAP + UKB) | Simple median | 4 | 1.005 (0.797 to 1.266) | 0.967 |
| Brassicasterol | AD (IGAP + UKB) | Weighted median | 4 | 0.854 (0.722 to 1.011) | 0.066 |
| Brassicasterol | AD (IGAP + UKB) | Weighted mode | 4 | 0.778 (0.658 to 0.919) | 0.060 |
| Brassicasterol | PD (IPDGC) | Inverse variance weighted | 4 | 0.988 (0.756 to 1.291) | 0.930 |
| Brassicasterol | PD (IPDGC) | Maximum likelihood | 4 | 0.988 (0.756 to 1.291) | 0.930 |
| Brassicasterol | PD (IPDGC) | MR Egger | 4 | 0.918 (0.589 to 1.431) | 0.743 |
| Brassicasterol | PD (IPDGC) | MR_PRESSO Outlier-corrected | 4 | NA (NA to NA) | NA |
| Brassicasterol | PD (IPDGC) | MR_PRESSO Raw | 4 | 0.988 (0.823 to 1.186) | 0.906 |
| Brassicasterol | PD (IPDGC) | Simple median | 4 | 0.960 (0.668 to 1.379) | 0.826 |
| Brassicasterol | PD (IPDGC) | Weighted median | 4 | 0.976 (0.735 to 1.295) | 0.866 |
| Brassicasterol | PD (IPDGC) | Weighted mode | 4 | 0.983 (0.733 to 1.317) | 0.914 |
| Brassicasterol | PD AAO (IPDGC) | Inverse variance weighted | 4 | 1.649 (0.282 to 9.658) | 0.579 |
| Brassicasterol | PD AAO (IPDGC) | Maximum likelihood | 4 | 1.652 (0.281 to 9.710) | 0.578 |
| Brassicasterol | PD AAO (IPDGC) | MR Egger | 4 | 5.399 (0.286 to 101.905) | 0.377 |
| Brassicasterol | PD AAO (IPDGC) | MR_PRESSO Outlier-corrected | 4 | NA (NA to NA) | NA |
| Brassicasterol | PD AAO (IPDGC) | MR_PRESSO Raw | 4 | 1.649 (0.414 to 6.575) | 0.529 |
| Brassicasterol | PD AAO (IPDGC) | Simple median | 4 | 0.697 (0.052 to 9.356) | 0.785 |
| Brassicasterol | PD AAO (IPDGC) | Weighted median | 4 | 1.906 (0.267 to 13.630) | 0.520 |
| Brassicasterol | PD AAO (IPDGC) | Weighted mode | 4 | 2.028 (0.295 to 13.934) | 0.524 |

Note: MR-PRESSO, Mendelian Randomization Pleiotropy RESidual Sum and Outlier; IVs, instrumental variables; N, number; NA, not applicable for exposure with less than 4 valid instrumental variables.


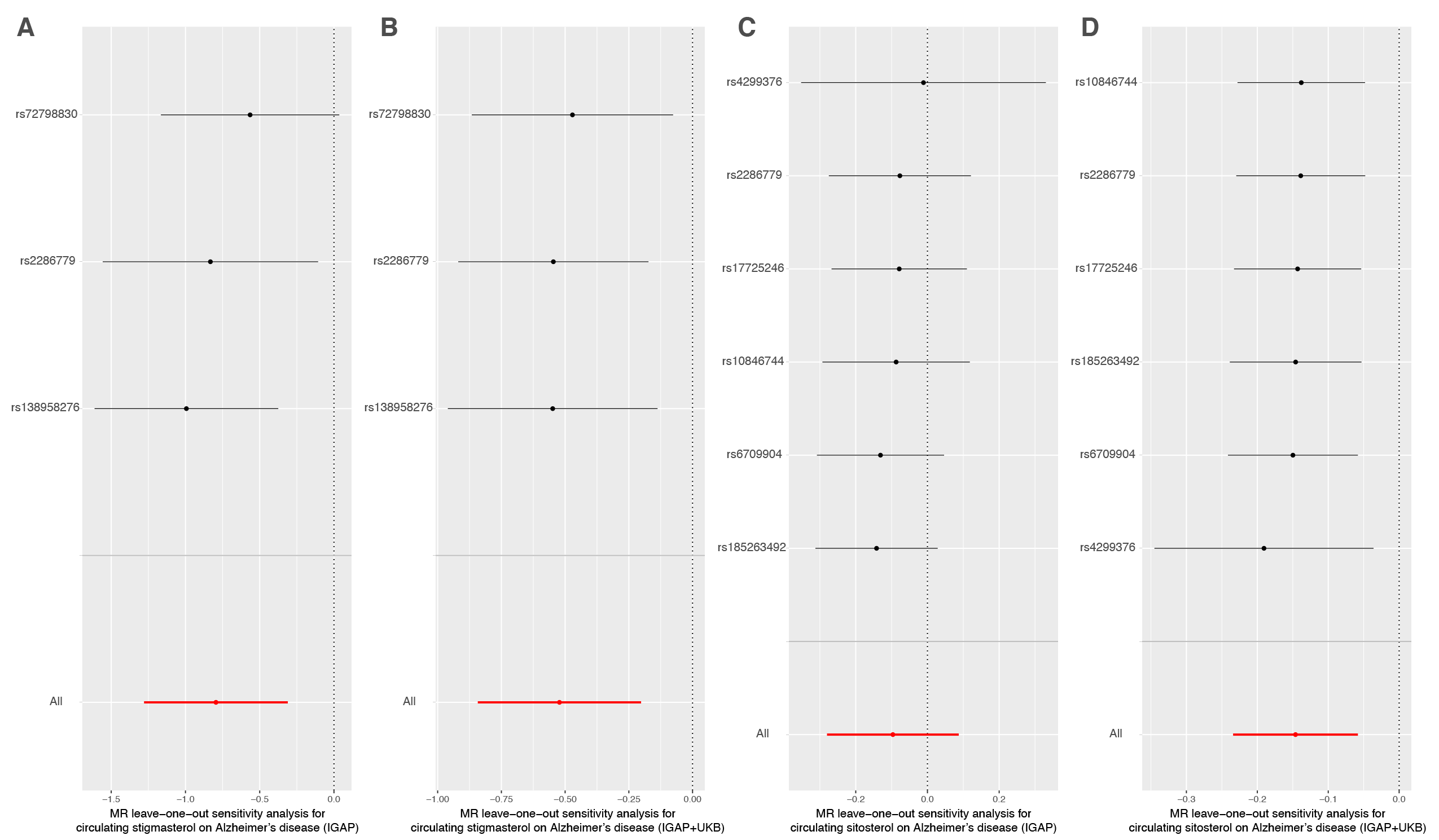


**Supplementary Figure S1. Leave-one-out plots for the causal effects of circulating levels of stigmasterol and sitosterol on the risk of Alzheimer’s disease.** A and B showed the leave-one-out plots for the causal effects of circulating stigmasterol levels on AD using IGAP and IGAP+UKB dataset individually. C and D showed the leave-one-out plots for the causal effects of circulating sitosterol levels on AD using IGAP and IGAP+UKB dataset respectively. AD, Alzheimer’s disease; IGAP, International Genomics of Alzheimer's Project; UKB, UK Biobank; MR, Mendelian randomization; SNP, single nucleotide polymorphism.
